# IL-17a-producing γδT cells and NKG2D signaling mediate bacterial endotoxin-induced neonatal lung injury: implications for bronchopulmonary dysplasia

**DOI:** 10.3389/fimmu.2023.1156842

**Published:** 2023-09-08

**Authors:** Tracy X. Cui, Alexander E. Brady, Ying-Jian Zhang, Chase Anderson, Antonia P. Popova

**Affiliations:** Department of Pediatrics, University of Michigan Medical School, Ann Arbor, MI, United States

**Keywords:** bronchopulmonary dysplasia (BPD), IL-17A, NKG2D, gamma delta T cells, inflammation, LPS, alveolar growth, prematurity

## Abstract

Bronchopulmonary dysplasia (BPD) is a chronic lung disease in preterm birth survivors characterized by inflammation, impaired alveolarization and dysmorphic vasculature. Activated IL-17A+ lymphocytes are key drivers of inflammation in preterm infants. We have shown that in immature mice chronic airway exposure to lipopolysaccharide (LPS) induces pulmonary inflammation, increased IL-17a expression, and hypoalveolarization, a BPD-like phenotype. The source of IL-17a and contribution to lung pathology is unknown. The natural-killer group 2, member D (NKG2D) receptor mediates activation and IL-17a production in γδ T cells by binding to stress molecules. LPS induces NKG2D ligand expression, including Rae-1 and MULT1. We hypothesized that IL-17a+ γδ T cells and NKG2D signaling mediate neonatal LPS-induced lung injury. Immature C57BL/6J (wild type), Nkg2d-/- or Tcrd-/- (lacking γδ T cells) mice were inoculated with 3ug/10ul of LPS from E. coli O26:B6 or 10ul of PBS intranasally on day of life 3, 5, 7, and 10. Selected mice were treated with neutralizing antibodies against IL-17a, or NKG2D intraperitoneally. Lung immune cells were assessed by flow cytometry and gene expression was analyzed by qPCR. Alveolar growth was assessed by lung morphometry. We established that anti-IL-17a antibody treatment attenuated LPS-induced hypoalveolarization. We found that LPS induced the fraction of IL-17a+NKG2D+ γδ T cells, a major source of IL-17a in the neonatal lung. LPS also induced lung mRNA expression of NKG2D, Rae-1, MULT1, and the DNA damage regulator p53. Anti-NKG2D treatment attenuated the effect of LPS on γδ T cell IL-17a expression, immune cell infiltration and hypoalveolarization. LPS-induced hypoalveolarization was also attenuated in Nkg2d-/- and Tcrd-/- mice. In tracheal aspirates of preterm infants IL-17A and its upstream regulator IL-23 were higher in infants who later developed BPD. Also, human ligands of NKG2D, MICA and MICB were present in the aspirates and MICA correlated with median FiO2. Our novel findings demonstrate a central role for activated IL-17a+ γδ T cells and NKG2D signaling in neonatal LPS-induced lung injury. Future studies will determine the role of NKG2D ligands and effectors, other NKG2D+ cells in early-life endotoxin-induced lung injury and inflammation with a long-term goal to understand how inflammation contributes to BPD pathogenesis.

## Introduction

Bronchopulmonary dysplasia (BPD), a common chronic lung disease in survivors of preterm birth, is associated with high long-term respiratory morbidity, increased healthcare use and expenditures (1–6). The pathology of BPD includes hypoalveolarization, interstitial thickening and dysmorphic vasculature (7, 8). These structural changes do not fully explain the chronic respiratory symptoms in these patients. Systemic and pulmonary inflammation precedes BPD development and persists through disease progression, but the driving cellular and molecular immune mechanisms are poorly understood, limiting the development of preventive and curative treatments (9–14).

Early life proinflammatory exposures, i.e., chorioamnionitis, sepsis and airway gram negative bacterial dominance are risk factors for BPD (15–21). Accumulating data implicate IL-17A-producing lymphocytes and activated nonconventional γδ T cells as key drivers of inflammation in preterm infants (22–27). Research with animal models of BPD by our and other groups has also previously shown that early life exposure to bacterial endotoxin induces pulmonary inflammation including increased IL-17a expression and characteristic lung pathology with impaired alveolar development (28–31). The specific roles of IL-17a, contribution of γδ T cells and signaling pathways that regulate these responses have not been elucidated.

IL-17a, a member of the IL-17 family, is a pro-inflammatory cytokine produced by T cells (T helper 17 cells), myeloid cells (neutrophils and macrophages), as well as innate lymphocytes, including γδ T cells and NK cells (32–35). IL-17a has pleiotropic functions in physiologic responses to limit fungal and bacterial infections (36, 37), and in the pathogenesis of acute and chronic inflammatory injury in the lungs and other organs (38–42). IL-17a also mediates pro-fibrotic lung responses (43, 44). Recent studies highlight additional roles for IL-17a-producing γδ T cells in maintaining local regulatory T cell homeostasis and thermogenesis (45). Furthermore, conditions promoting oxidative metabolism during development direct γδ T cells to IL-17-producing fate (46). γδ T cells are the main source of IL-17a in mucosal tissues, including in the lung (47, 48). γδ T cells participate in the innate immune response to bacterial endotoxin directly via TLR4 on their surface, which results in increased IL-17a production (49). Bacterial endotoxin also induces cellular stress and increased expression of ligands for activating receptors, including natural-killer group 2, member D (NKG2D) (50). When activated, NKG2D induces pro-inflammatory IL-17a responses (51–53).

In this study, we hypothesized that IL-17a-producing γδ T cells and NKG2D signaling mediate early-life bacterial endotoxin-induced pulmonary inflammation and impaired alveolar development.

## Methods

### Study approval

The human study was approved by the University of Michigan Institutional Review Board. All mouse experiments were performed following the NIH Guide for the Care and Use of Laboratory Animals recommendations. The animal protocol was approved by the University of Michigan Committee on Use and Care of Animals.

### Human tracheal aspirate collection, multiplex measurement, and qPCR

We examined tracheal aspirates from infants admitted to the C.S. Mott Children’s Hospital Newborn Intensive Care Unit. Entry criteria included gestational age at birth ≤ 32 weeks, mechanical ventilation for respiratory distress, and age ≤ 7 days. Aspirates were collected during routine tracheal suctioning of mechanically ventilated premature infants as described (54). Patient demographics are included in **Table 1**. Human IL-17A and IL-23 were measured using the tracheal aspirate supernatants with a customized Multiplex ELISA (MilliporeSigma, Burlington, MA). A 200 μl aliquot of the tracheal aspirate was stored in RLT buffer (Qiagen, Valencia, CA) at -80°C. Total RNA was extracted using the RNeasy Plus Micro kit (Qiagen). MHC class-I-related protein A (MICA) AND MICB mRNA expression was determined by qPCR. Median FiO2 required between birth and the day of sample collection for each infant was recorded.

**Table 1:** Baseline characteristics of infants who developed BPD or died before 36 weeks postmenstrual age (BPD/death) and infants who did not develop BPD (No BPD).

	BPD/death (N=37)	No BPD (N=17)	P-value
Male gender, n (% total)	23 (62)	12 (71)	0.76
Gestational Age, wks, mean ± SD	25.9 ± 1.7	29.4 ± 1.7	<0.0001
Birth weight, grams, mean ± SD	871 ± 301	1361 ± 308	<0.0001
Postnatal Age at Sample Collection, days, median (IQR)	3 (3)	2 (2)	<0.05
Suspected clinical chorioamnionitis, n (% total)	9 (24)	1 (6)	0.14
Antenatal steroids, n (% total)	26 (70)	9 (53)	0.24
Vent days, median (IQR)	47 (52)	5 (9.5)	<0.0001
O2 days, median (IQR)	216 (245.5)	24 (37.25)	<0.0001

IQR, interquartile range; Vent days, days requiring mechanical ventilations; O2 days, days requiring supplemental oxygen. Gestational age and birth weight were compared using Student’s t-test. Postnatal age at sample collection, vent days and O2 days were compared using Wilcoxon-Mann-Whitney test. Gender, frequency of MSCs, suspected clinical chorioamnionitis, antenatal steroids were compared using Fisher’s exact test.

### Animal model

Neonatal C57BL/6J (wild type), Klrk1-/- (B6.Cg-Klrk1^tm1Dhr^/J, Nkg2d-/-) and Tcrd-/- (B6.129P2-Tcrd^tm1Mom^/J) mice were purchased from Jackson Laboratories (Bar Harbor, ME). Wild type and knock out mice were inoculated with 3μg/10μl of LPS from E. coli O26:B6 (Sigma-Aldrich, St. Louis, MO), or endotoxin-free PBS (Sigma-Aldrich), intranasally on day of life (DOL) 3, 5, 7, and 10 as described (28). Selected mice were injected intraperitoneally with 0.5 mg/kg of anti-IL17a antibody (clone TC11-18H10, BioLegend, San Diego, CA) or an IgG control (Rat IgG control, BioXCell, Lebanon, NH) on DOL 3, 4, 5, 6, 7, 10 and 12. Some mice were injected intraperitoneally with anti-NKG2D antibody (Clone HMG2D) or IgG control (10mg/kg) (BioXCell) 1 hr prior to each LPS treatment.

### Quantitative real-time PCR

Mouse whole lung tissues were harvested in TRIzol (Zymo Research, Irvine, CA) and homogenized on ice. RNA extraction was performed according to Direct-zol™ RNA MiniPrep (Zymo Research, Irvine, CA) manufacturer instructions. Complementary DNA was synthesized following the TaqMan Reverse Transcription Reagents protocol (Life Technologies, Carlsbad, CA). The expression levels of genes of interest were quantified with SYBR Green technology. The specific primer sequences are available upon request. Relative gene expression was analyzed with the 2−ΔCT algorithm by normalizing the level of gene expression for each sample to glyceraldehyde 3-phosphate dehydrogenase (GAPDH) and beta-actin (β-Actin) as indicated.

### Flow cytometry analysis

Lungs were perfused with PBS containing EDTA (0.5 mM), minced, and digested with Liberase TM (100 µg/mL; Roche, Indianapolis, IN), together with collagenase XI (250 µg/mL), hyaluronidase 1a (1 mg/mL), and DNase I (200 µg/mL; Sigma, St. Louis, MO) for 1 hour at 37°C (55). Cells were filtered and treated with RBC lysis buffer (BD Biosciences, Franklin Lakes NJ) and kept on ice in media containing 10% serum. Dead cells were stained with Pac-Orange Live/Dead fixable, dead staining dye (ThermoFisher Scientific). Cells were incubated with Protein Transportation Inhibitor cocktail (Brefeldin A and Monensin) (ThermoFisher Scientific, Waltham,MA) for 3 hr. Lung cells were then stained with fluorescently labeled antibodies against various leukocyte surface markers (CD45, F4/80, NK1.1, CD3ϵ, TCRβ, TCRγδ, NKG2D (clone CX5), IL-17a and IFNγ). Appropriate isotype-matched controls and Fluorescence Minus One (FMO) controls were used in all experiments. Antibodies were purchased from eBiosciences (San Diego, CA) or Biolegend (San Diego, CA). Cells were fixed and analyzed on a Fortessa (Becton-Dickinson, San Jose, CA) or FACSAria II (BD Biosciences) flow cytometer. Results were analyzed using FlowJo software (Tree Star, Ashland, OR).

### Lung histology and morphometry

Lungs were perfused with 5 mM EDTA, inflated to 30 cmH2O pressure with 4% paraformaldehyde (Sigma-Aldrich, St. Louis, MO), and paraffin embedded. Five μm-thick paraffin sections were stained with Hematoxylin and eosin (H&E). Images were acquired using Olympus IX71 microscope (Tokyo, Japan). To assess alveolarization, alveolar chord length was measured as described (56, 57). In summary, alveolar chord length was calculated as the mean length of line segments on random test lines spanning the airspace between intersections of the line with the alveolar surface was calculated. Four random images from two sections for each animal were photographed at X200.

### Statistical analysis

All data were described as mean ± SEM or median and interquartile range as appropriate. An unpaired t-test, Mann-Whitney test or one-way ANOVA test were used for comparison. P values were considered statistically significant if they were < 0.05.

## Results

### Pro-inflammatory cytokine IL-17A and its upstream regulator IL-23 are elevated in tracheal aspirates of premature infants with respiratory distress who go on to develop BPD

**Figure 1:**
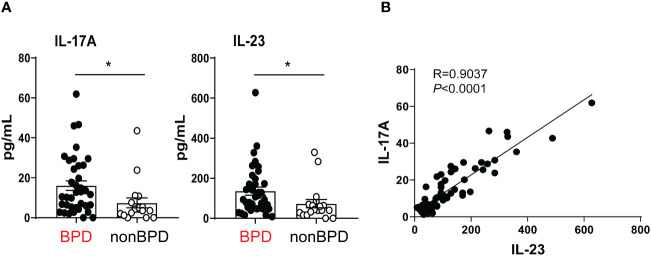
Inflammatory cytokines IL-17A and IL-23 are elevated in the tracheal aspirates of premature infants who later develop BPD. Mechanically ventilated premature infants without other comorbidities were recruited to the study. Tracheal aspirates were collected during the first week of life. **(A)** IL-17A and IL-23 protein levels were detected by Multiplex ELISA in the supernatants of the tracheal aspirates. BPD diagnosis was recorded based on chart review once the subjects reached 36 weeks postmenstrual age. Because death is a competing outcome for BPD, infants who died before 36 weeks postmonstrual age were included in the BPD/death group. This group included 37 infants. 17 infants did not develop BPD (No BPD). Both IL-17A and IL23 protein levels were elevated in infants who later developed BPD compared to infants who did not go on to develop BPD. *p<0.05 (unpaired *t*-test). **(B)** IL-23 protein levels significantly correlated with IL-17A levels. Pearson’s correlation analysis. R=0.9037, *p*<0.0001.

Prior human studies implicate IL-17A-producing lymphocytes as key drivers of inflammation in preterm infants (22, 23). The pattern of IL-17A expression in the airways or preterm infants at risk for BPD development is unknown. We tested if levels of IL-17A and its upstream regulator IL-23 are increased during the first week of life in the airways of premature infants who go on to develop BPD. We measured IL-17A and IL-23 in tracheal aspirates from human preterm infants mechanically ventilated for respiratory distress in the first week of life. Patient characteristics are described in **Table 1**. We defined BPD based on the need for supplemental oxygen at 36 weeks postmenstrual age. For prematurely born infants, death before 36 weeks postmenstrual age is a competing outcome for BPD and may confound a cause-specific analysis, therefore we combined both outcomes. We found that tracheal aspirates from premature infants who go on to develop BPD have significantly higher IL-17A and IL-23 protein levels compared to aspirates from infants who do not develop BPD (**Figure 1A**). Additionally, IL-17A protein levels positively correlated with levels of IL-23 (**Figure 1B**). These results suggest that higher levels of proinflammatory cytokines IL-17A and IL-23 are associated with BPD diagnosis, a predictor of higher chronic respiratory morbidity. These novel findings implicate IL-17A-driven inflammation in BPD pathogenesis.

### *In vivo* blockade of IL-17a attenuated chronic bacterial endotoxin-induced hypoalveolarization in experimental mouse model of BPD

**Figure 2:**
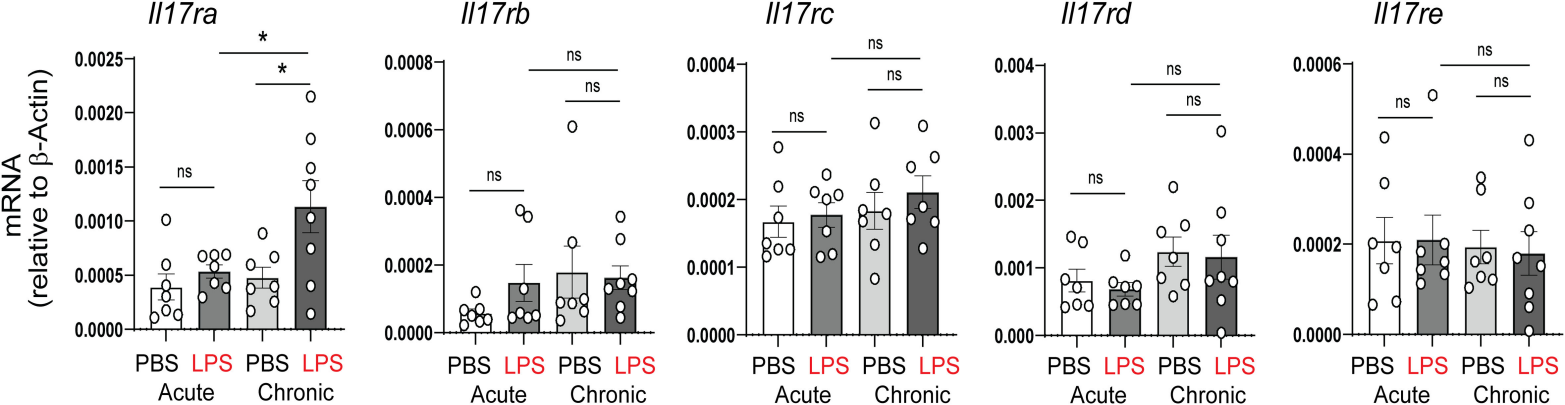
Effects of neonatal LPS exposure on IL-17 receptor expression. Immature C57BL/6J mice were inoculated with LPS or PBS intranasally on DOL 3, 5, 7 and 10. Lungs were harvested after acute (one day after the first dose) and chronic (one day after the last dose) LPS exposure. mRNA expression of the IL-17 family receptors, Il17ra, Il17rb, Il17rc, Il17rd, and Il17re was quantified by qPCR (N=7-8 neonatal mice per group from at least two different experiments, mean±SEM, *P<0.05, one-way ANOVA. ns, none-significant.

In our previous study, acute and chronic neonatal LPS exposure induced pulmonary inflammation and ultimately resulted in persistent hypoalveolarization (28). Among the proinflammatory cytokines upregulated by neonatal LPS were TNF-α, IL-6 and IL-17a. To further assess the role of IL-17a/IL-17ra pathway in this model, we examined the mRNA expression of the IL-17a receptor, IL-17ra, as well as the expression of other members of the IL-17 receptor family, including, IL-17rb, IL-17rc, IL-17rd, and IL-17re. There were no significant changes in any IL-17 receptors after acute LPS. Chronic LPS induced the expression of IL-17ra and did not affect the expression of the other IL-17 receptor family members (**Figure 2**).

**Figure 3:**
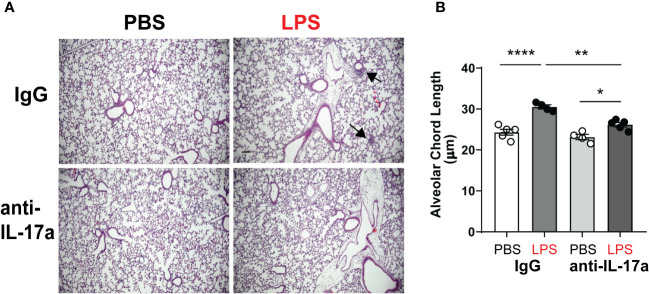
*In vivo* blockade of IL-17a with IL17a neutralizing antibody attenuated chronic neonatal LPS-induced hypoalveolarization. Immature C57BL/6J mice were inoculated with LPS or PBS intranasally on DOL 3, 5, 7 and 10. Anti-IL-17a antibody or IgG control were injected intraperitoneally on DOL 3,4,5,6,7,10 and 12. Lung tissues were inflated, fixed, and embedded in paraffin on DOL14. After H&E staining, lungs were examined by light microscopy and lung morphometry analysis quantified alveolar chord lengths as measures of alveolar growth. In the IgG-treated mice, inoculation with LPS led to immune cell infiltration (black arrows) and increased alveolar size indicative of alveolar simplification **(A)**. **(B)** Chronic LPS inoculation of IgG-treated mice significantly increased alveolar chord length. Anti-IL17a antibody treatment attenuated LPS-induced immune cell infiltration and hypoalveolarization, as measured by alveolar chord length. **p<0.01, *p<0.05 (one-way ANOVA). Each open or black circle indicates one baby mouse. N=4-5 mice per group for both histological evaluation and chord length measurements. Scale bar (black) indicates100mm. ****p<0.0001.

To test the requirement of IL-17a for LPS induced hypoalveolarization, we used a neutralizing antibody against IL-17a (58). Immature wild type mice were inoculated with LPS or PBS and treated with anti-IL-17a antibody, administered intraperitoneally in multiple doses as described above. Subsequently, we examined lung histology on DOL14. As expected, chronic LPS exposure induced larger and fewer alveolar spaces resulting in increased alveolar chord lengths (**Figure 3**). In contrast, the alveolar size of LPS-exposed anti-IL-17a antibody treated mice appeared similar to the PBS-treated mice (**Figure 3A**). Among the LPS-exposed neonatal mice, the alveolar chord lengths of anti-IL-17a-treated mice were significantly smaller compared to IgG-treated mice (**Figure 3B**). These results indicate that *in vivo* blockade of IL-17a protects from LPS-induced BPD-like lung pathology.

### γδ T cells are the major source of IL-17a during acute and chronic neonatal LPS exposure

**Figure 4:**
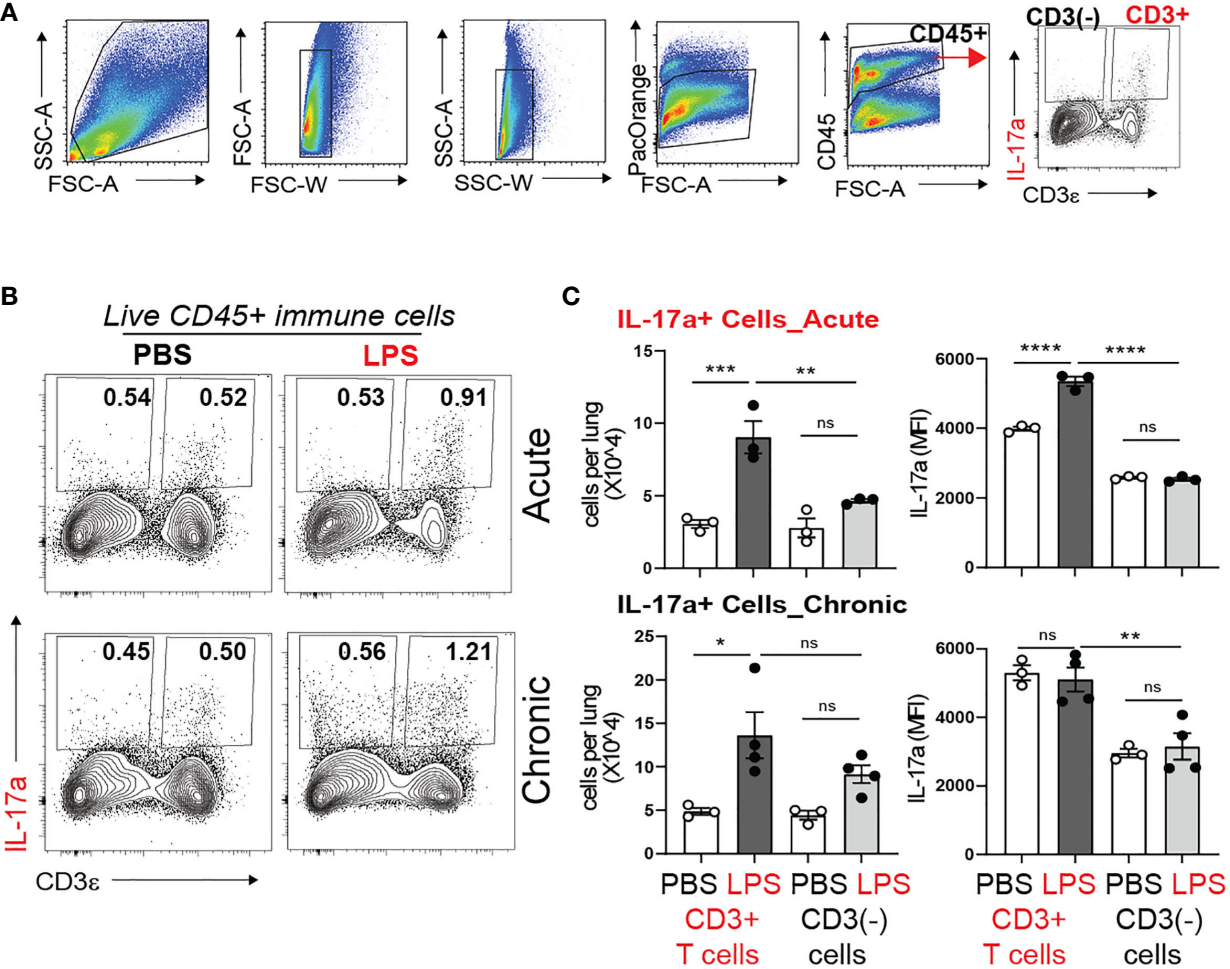
Acute and chronic LPS-induced IL-17a+ CD3+ T cells are the major IL-17a expressing immune cells in the neonatal lung. Immature C57BL/6J mice were inoculated with LPS (L) or PBS (P) intranasally on DOL 3, 5, 7, and 10. Lungs were analyzed during acute (one day after 1 dose) and chronic (one day after 4 doses) exposure. Lung digests were subjected to flow cytometry. **(A)** Flow gating strategy for detection of IL-17a+ immune cells in the neonatal lung. After acute and chronic LPS (L) exposure there was a significant increase in the fraction **(B)** and total number **(C)** of IL-17a+CD3+T cells. **(C)** Acute LPS exposure also increased the per cell expression of IL-17a among the CD3+ T cells compared with PBS (P) controls. In contrast, LPS did not induce IL-17 expression in non-CD3 immune cells. *P<0.05, ns, non-significant (one-way ANOVA). Each open or black circle indicates one baby mouse. N=3-4 per group. One of at least two independent experiments is presented. **p<0.01, ***p<0.001, **** p<0.0001.

During late gestation and early postnatal life the developing lung is populated with resident innate immune cells, including macrophages and lymphocytes (59–61). There is paucity of data what cells produce IL-17a in response to bacterial endotoxin during the first weeks of life. To determine the cellular source of IL-17a, we examined lungs by flow cytometry one day after one dose of LPS (acute LPS exposure) and one day after four doses of LPS (chronic LPS exposure). **Figure 4A** presents the gating strategy. We divided the population of Live CD45+ immune cells into CD3+ T cells and non CD3 immune cells (CD3(-) immune cells) and analyzed the effect of LPS on the constitutive IL-17a production in these two populations (**Figure 4B**). Acute and chronic LPS exposure significantly increased the number of IL-17a-producing CD3+T cells but not CD3(-) immune cells (**Figure 4C**). Acute LPS exposure also significantly increased the average per cell IL-17a protein expression in CD3+ T cells presented as Median Fluorescence Intensity (MFI). These results demonstrate that CD3+T cells are the main source of IL-17a after acute and chronic neonatal LPS exposure.

**Figure 5:**
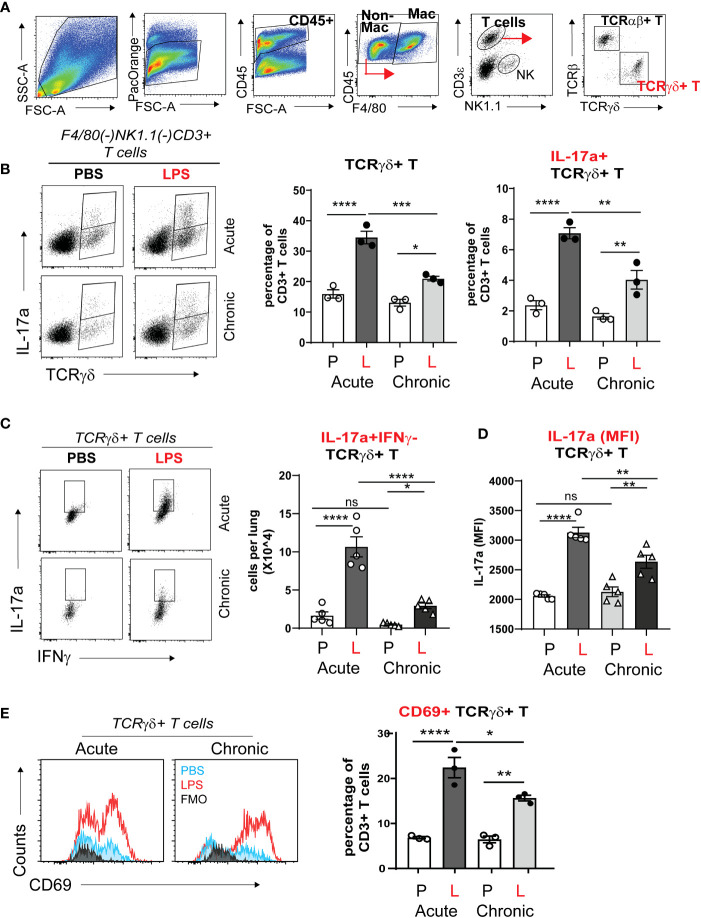
TCRγδ+ T cells but not TCRαβ+ T cells express IL-17a in neonatal lung in response to LPS. Immature C57BL/6J mice were inoculated with LPS (L) or PBS (P) intranasally on DOL 3, 5, 7, and 10. Lungs were analyzed during acute (one day after 1 dose) and chronic (one day after 4 doses) exposure. Lung digests were subjected to flow cytometry. **(A)** Gating strategy for detection of TCRγδ+ T cells and TCRαβ+ T cells. The γδ+ T cells are identified by flow cytometry sequential analysis as LiveCD45+F4/80(-)Nk1.1(-) CD3ε+TCRβ(-) TCRγδ+ T cells. **(B)** After Acute and Chronic LPS exposure there was a significant increase in the fraction of IL-17a expressing TCRγδ+ T cells but not TCRαβ+ T cells. **(C)** LPS did not induce IFN-γ expression in TCRγδ+ T cells. **(D)** LPS increased the per cell expression of IL-17a among the TCRγδ+ T cells presented as IL-17a (MFI). **(E)** LPS-induced TCRγδ+ T cells express CD69 activation and tissue resident marker. *P<0.05, **P<0.01, ****P<0.0001, ns, non-significant (one-way ANOVA). Each open or black circle indicates one baby mouse. N=3-5 per group. One of at least two independent experiments is presented. ***p<0.001.

To further define the CD3+ T cell populations responsible for IL-17a production after acute and chronic neonatal LPS exposure, we differentiated the CD3+ T cells into TCRαβ+ T cells and TCRγδ+ T cells and analyzed their constitutive IL-17 production using flow cytometry analysis and gating on LiveCD45+F4/80(-)NK1.1(-)CD3e+ cells (**Figure 5A**). The fraction of γδ T cells in the adult mouse lung is less than 10% of CD3+ T cells at baseline and increases several folds upon inflammatory stimulation (62). We found that in the neonatal mouse lung at baseline, the fraction of γδ T cells is about 15% of the total CD3+T cells on DOL4 and DOL10 PBS exposure (**Figure 5B**). This is slightly higher compared to the fraction reported in adult mice. Acute and chronic neonatal LPS exposure significantly increased the fraction of γδ T cells (**Figure 5B**). After acute LPS exposure, γδ T cells increased to 35% of the total CD3+ T. Both acute and chronic LPS exposure also significantly increased the fraction of γδ T cells that constitutively expressed IL-17a in the neonatal lung (**Figure 5B**). The increase was greater in acute compared to chronic LPS exposure. IL17a was almost exclusively produced by γδ T cell and not by TCRγδ(-) T cell populations. A small fraction (2%) of γδ T cells produced IL-17a at baseline (**Figure 5B**). Acute and chronic LPS exposure induced higher per cell expression of IL-17a (MFI) in γδ T cells (**Figure 5D**). The LPS-induced IL-17a+ γδ T cells did not produce IFN-γ (**Figure 5C**) but expressed CD69 (**Figure 5E**) consistent with activated tissue-resident phenotype. To our knowledge, this is the first report describing increased IL-17a-producing γδ T cells in neonatal lungs after LPS exposure, a mouse model of BPD. These results indicate that γδ T cells are the major source of IL-17a in the neonatal lung after acute and chronic LPS exposure. These findings implicate activated IL-17a+ γδ T cells in BPD pathogenesis.

### Neonatal LPS exposure induces the expression of the activating receptor NKG2D and its stress response ligands

**Figure 6:**
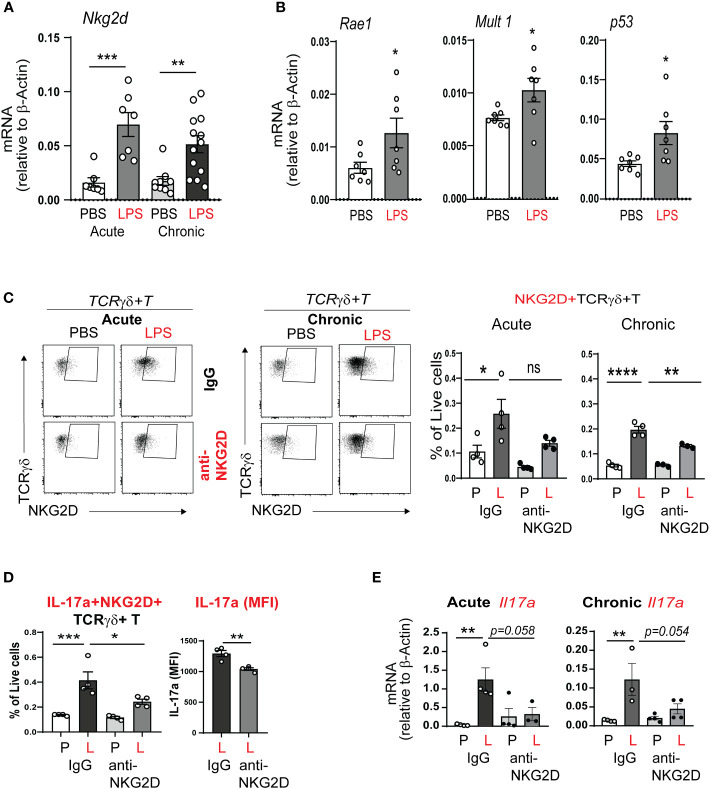
NKG2D mediates acute and chronic neonatal LPS-induced γδ T cell activation and IL-17a production. **(A)** Nkg2d mRNA expression was increased in neonatal lung after acute and chronic exposure to LPS. **(B)** LPS induced neonatal lung mRNA expression of the NKG2D ligands Rae1 and Mult1, as well as the master regulator of DNA damage p53. **(C, D)** We inoculated immature C57BL/6J mice with LPS or PBS intranasally on DOL 3, 5, 7 and 10. One hour prior to each PBS or LPS treatment, mice were injected with IgG or anti-NKG2D antibody (Clone HMG2D) intraperitoneally. Lungs were examined by flow cytometry after acute and chronic LPS exposure. The NKG2D receptor expression, as a marker of activation, was detected on γδ T cells using a different clone of the anti-mouse NKG2D antibody (clone CX5). NKG2D+ γδ T cells were increased after acute and chronic LPS treatment in the neonatal lung, consistent with γδ T cell activation. IL-17a+NKG2D+ γδ T cells were also increased. Anti-NKG2D antibody treatment attenuated the effects of LPS on the activated NKG2D+ γδ T cells and the fraction of these cells that produced IL-17a. This analysis quantified native IL-17a expression only using a Protein Transportation Inhibitors cocktail. Per cell IL-17a expression (MFI) in NKG2D+ γδ T cells after LPS exposure was quantified and the effect of *in vivo* anti-NKG2D treatment was assessed. **(E)** Both acute and chronic LPS exposure induced whole lung *Il17a* mRNA expression and this effect was inhibited with *in vivo* anti-NKG2D treatment. *P<0.05, **P<0.01, ***P<0.001, ****P<0.0001, ns – non-significant (one-way ANOVA and unpaired *t* test). Each open circle indicates one baby mouse. One of at least two independent experiments is presented.

Since acute neonatal lung LPS exposure quickly induced IL-17a production in γδ T cells, we hypothesized that this process is regulated by a conserved innate immune response. The activating receptor NKG2D responds to cellular stress by binding to its ligands and promotes inflammation and cytotoxicity (51, 53). We examined the effects of neonatal LPS exposure on whole lung NKG2D mRNA expression. We found that both acute and chronic neonatal LPS exposure significantly increased whole lung NKG2D mRNA expression (**Figure 6A**). NKG2D ligands in mice include retinoic acid early inducible-1 (Rae-1), murine ULBP-like transcript 1 (MULT1), and others (63, 64). We found that neonatal mice exposed to LPS show induced whole lung expression of the NKG2D ligands Rae-1, MULT1 and the expression of the DNA damage factor p53 (**Figure 6B**). Together these results demonstrate that LPS exposure induces the expression of the activating NKG2D receptor and its stress response ligands. These findings indicate NKG2D signaling contributes to BPD immunopathology.

### Contribution of NKG2D signaling to lung γδ T cells responses to acute and chronic neonatal LPS exposure

Next, we hypothesized that NKG2D signaling mediates neonatal LPS-induced IL17a production in lung γδ T cells. To test our hypothesis, immature wild type mice were inoculated with LPS or PBS intranasally on DOL 3, 5, 7 and 10. One hour prior to each LPS or PBS treatment the mice were treated with anti-NKG2D antibody or IgG control intraperitoneally. The lungs were analyzed after acute and chronic LPS exposure. Lung immune cells were subjected to flow cytometry analysis to detect NKG2D protein expression on target immune cells. The blocking mAb and FACS mAb that we used target different epitope regions of NKG2D, allowing us to prevent competitive binding to the receptor. NKG2D protein expression was observed on the surface of γδ T cells (**Figure 6C**). The fraction of NKG2D+ γδ T cells was increased in the neonatal lung after acute and chronic LPS treatment (**Figure 6C**). Anti-NKG2D treatment significantly reduced the fraction of NKG2D+ γδ T cells (**Figure 6C**). Further, IL-17a+NKG2D+ double positive γδ T cells were increased after LPS, and the fraction of these cells was attenuated by anti-NKD2D treatment (**Figure 6D**). Anti-NKG2D also reduced the LPS-induced per cell expression of IL-17a. In addition, anti-NKG2D treatment attenuated acute and chronic LPS exposure-induced IL-17a mRNA expression (**Figure 6E**). To our knowledge, this is the first report to show that neonatal LPS exposure stimulates NKG2D expression on γδ T cells and that blocking NKG2D reduces LPS-induced pro-inflammatory IL-17a response in NKG2D+ γδ T cells.

**Figure 7:**
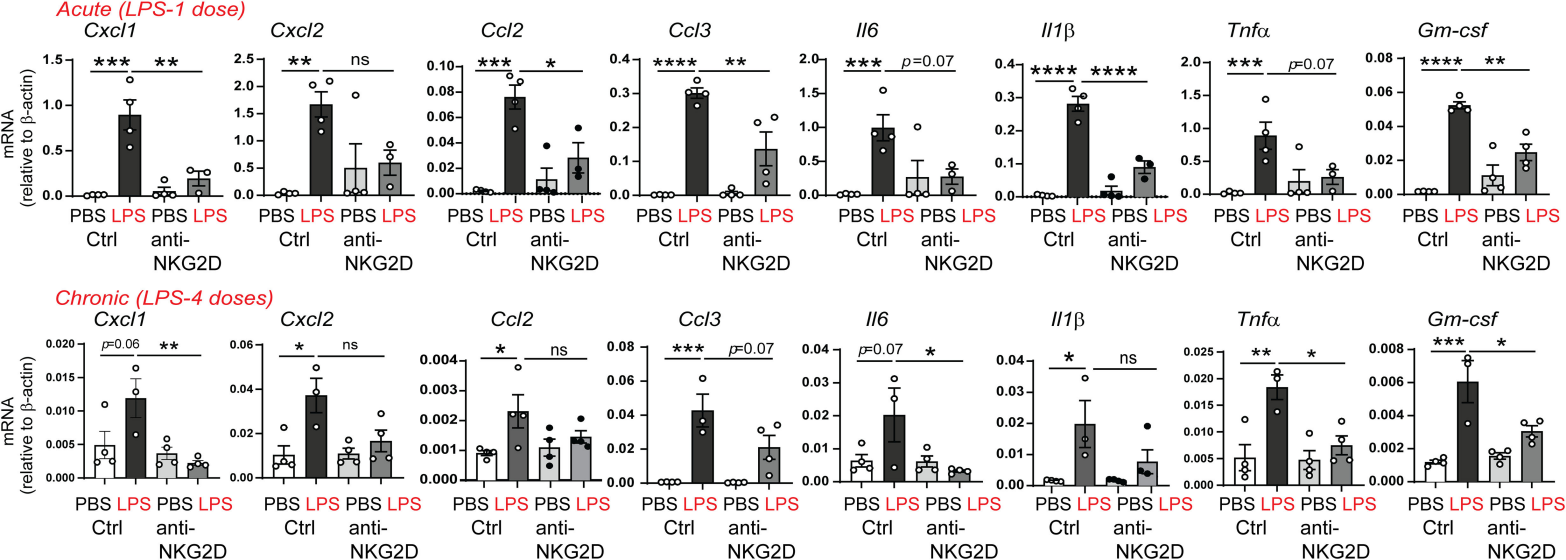
NKG2D blockade attenuates acute and chronic LPS-induced pro-inflammatory chemokine and cytokine expression. Immature mice were inoculated with LPS intranasally and injected with IgG or anti-NKG2D antibody as described above. Lungs were harvested after acute and chronic LPS exposure. mRNA expression of CXCL1, CXCL2, CCL2, CCL3, IL-6, IL-1β, TNF-α, and GM-CSF was quantified by qPCR (N=3-4 neonatal mice per group, one of at two independent experiments is presented, mean±SEM, *P<0.05, **P<0.01, ***P<0.001, ****P<0.0001, ns, non-significant, one-way ANOVA).

To assess the effect of NKG2D blockade on the neonatal lung capacity to recruit inflammatory cells in response to LPS, we examined the expression of the neutrophil chemoattractants CXCL1, CXCL2, monocyte chemoattractants CCL2, CCL3, proinflammatory IL-6, the M1 macrophage markers IL-1β, and TNF-α, as well as the M1 macrophage differentiation factor GM-CSF. We found that anti-NKG2D antibody treatment attenuated both acute and chronic LPS-induced mRNA expression of these chemokines and cytokines (**Figure 7**). These results indicate that NKG2D blockade reduced the capacity to recruit neutrophils and macrophages and attenuated the LPS-induced inflammatory milieu in the neonatal lungs.

### NKG2D and γδ T cells are required for chronic LPS-induced hypoalveolarization

**Figure 8:**
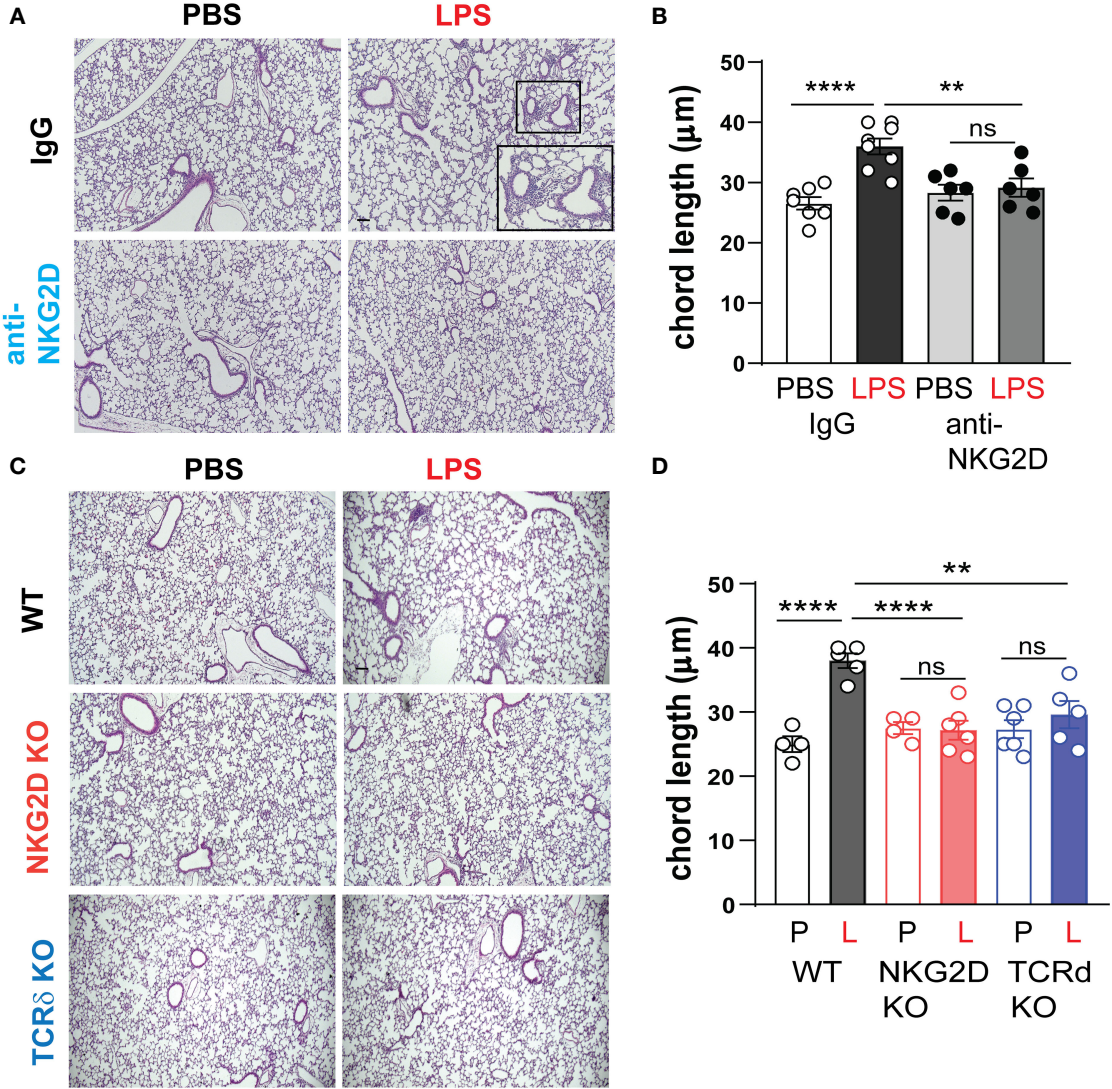
NKG2D and γδ T cells are required for chronic LPS-induced hypoalveolarization. **(A)** Immature C57BL/6J mice were inoculated with LPS or PBS intranasally on DOL 3, 5, 7, and 10. One hour prior to each PBS/LPS treatment, mice were injected with IgG or anti-NKG2D antibody (Clone HMG2D) intraperitoneally. Lung histology was analyzed on DOL14. The representative images of H&E-stained lung sections are shown. In the IgG-treated lungs, LPS induced peribronchial or perivascular immune cell infiltration and increased alveolar size **(A)** with increased chord length **(B)**, indicative of alveolar growth deficiency. Scale bar (black) indicates100μm. Anti-NKG2D antibody treatment blocked LPS-induced immune cell infiltration and enlarged alveolar size in the lungs, consistent with protective effects on lung inflammation and alveolarization. **(C)** To further define the requirement of NKG2D and γδ T cells for LPS-induced hypoalveolarization, we inoculated immature wild type, Nkg2d -/- or Tcrd-/- mice with LPS (L) or PBS (P) intranasally on DOL 3, 5, 7, and 10. Lung histology was analyzed on DOL14. Unlike wild type mice, Nkg2d -/- and Tcrd-/- mice did not develop immune cell infiltration or increased alveolar size after LPS. **(D)** The chord length measurement showed that LPS-induced hypoalveolarization (increased chord length) was blocked in Nkg2d -/- or Tcrd-/- mice. **P<0.01, ****P<0.0001, ns, non-significant (one-way ANOVA). Each open or black circle indicates one baby mouse. N=4-8 per group. Scale bar (black) indicates100μm.

We inoculated immature wild type mice LPS or PBS intranasally on DOL 3, 5, 7 and 10. One hour prior to each LPS or PBS treatment, the mice were injected intraperitoneally with anti-NKG2D antibody or IgG control. Lung histological analysis assessed immune cell infiltration and the degree of alveolar growth impairment on DOL 14. We found that in IgG-treated mice, LPS-induced immune cell infiltration and larger alveoli, consistent with hypoalveolarization and the LPS effect was significantly reduced in anti-NKG2D-treated lungs (**Figure 8A**). Alveolar chord length measurements quantified the differences in alveolarization and showed a similar pattern (**Figure 8B**). In a second experiment, we assessed the effect of chronic LPS exposure on lung inflammatory cell infiltration and alveolarization in Nkg2d-/- and Tcrd-/- mice and compared them to wild type mice. Unlike wild type mice, neither Nkg2d-/- mice nor Tcrd-/- mice developed inflammatory cell infiltration or hypoalveolarization after chronic LPS exposure (**Figures 8C, D**). These results indicate that NKG2D signaling and γδ T cells are required for the effects of chronic LPS on alveolarization.

### Human NKG2D ligands MICA and MICB mRNAs are present in tracheal aspirates from preterm infants in the first week of life, and ICA positively correlates with median FiO2

**Figure 9:**
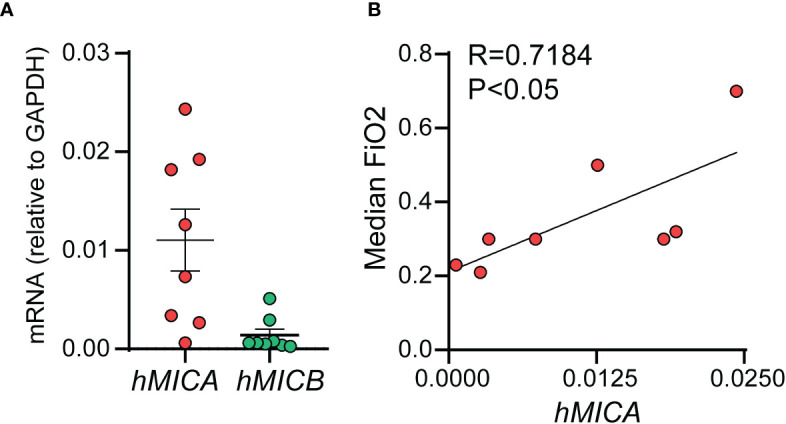
mRNAs of human NKG2D ligands MICA and MICB were present in tracheal aspirates collected from preterm infants mechanically ventilated for respiratory distress in the first week of life. RNA was prepared from the infant tracheal aspirate samples. MICA mRNA expression was higher than MICB mRNA expression **(A)**. MICA mRNA expression positively correlated with median FiO2 required by each subject up until the day of sample collection **(B)**, Pearson correlation.

We examined the mRNA expression of the human NKG2D ligands MICA and MICB in tracheal aspirates from human premature infants mechanically ventilated for respiratory distress in the first week of life (**Figure 9**). MICA mRNA expression was higher than MICB mRNA expression (**Figure 9A**). Further, MICA mRNA expression was positively correlated with median FiO2 required by each subject between birth and the day of sample collection (**Figure 9B**). These results indicate that cellular stress signals that bind to NKG2D are present in the airways of premature infants during the first week of life and may contribute to BPD pathogenesis by NKG2D-dependent pro-inflammatory activation of innate immune cells.

## Discussion

To examine the effect of early-life pro-inflammatory exposures on pulmonary inflammation and lung alveolar development, we developed a mouse model of neonatal exposure to LPS, a Gram-negative bacteria-derived immunogen. Chronic neonatal lung LPS exposure induces pulmonary inflammation, increases pro-inflammatory cytokine expression, including IL-17a expression, and results in hypoalveolarization (28). While the LPS-induced histopathology is reminiscent of human BPD, little is known about the role of IL-17a in BPD development. In this study, we investigated the contribution of a novel inflammatory pathway driven by IL-17a-producing γδ T cells and the activating NKG2D signaling in BPD pathogenesis. We discovered that neonatal lung NKG2D+ γδ T cells are the major source of IL-17a after acute and chronic neonatal LPS exposure. Neonatal LPS exposure also induced whole lung NKG2D gene expression and NKG2D ligand expression (Rae-1, MULT1). NKG2D blockade attenuated the effects of LPS on NKG2D+ γδ T cells and IL-17a expression, and inhibited LPS-induced immune cell infiltration and hypoalveolarization. We observed similar protective effects in Nkg2d-/- and Tcrd-/- mice. Finally, we demonstrated that tracheal aspirates of premature infants who were mechanically ventilated in the first week of life for respiratory distress showed higher levels of IL-17A and its upstream regulator IL-23 in infants who go on to develop BPD compared to infants who do not develop BPD. The tracheal aspirates also contained mRNAs of the human NKG2D ligands MICA and MICB, and MICA mRNA expression levels positively correlated with median FiO2, suggesting a link between oxygen exposure and stress responses. Together, our findings highlight an essential role for IL-17a-producing γδ T cells and NKG2D signaling in bacterial endotoxin-induced neonatal lung injury and in BPD pathogenesis.

There is ample evidence that early-life infection or inflammation, i.e., chorioamnionitis, sepsis and gram-negative bacterial dominance in airways are risk factors for BPD (16–20, 65). While prior research supports roles for IL-17A+ lymphocytes and activated γδ T cells as key drivers of inflammation in preterm infants (22–26), little is known about the contribution of IL-17a-producing cells in BPD pathogenesis. Cord blood of infants who were prenatally exposed to chorioamnionitis, a risk factor for BPD, contains higher frequency of IL-17a-expressing T lymphocytes (23). Moreover, in experimental model of chorioamnionitis, maternal exposure to LPS increases IL-17a expression in the offspring lung T lymphocytes (31). Additionally, IL-17a has been implicated in pro-fibrotic responses and development of pulmonary fibrosis (44, 66–68). IL-17A levels are increased in BAL of patients with idiopathic pulmonary fibrosis (43). To our knowledge, this is the first report demonstrating increased IL-17A levels in tracheal aspirates from premature infants with respiratory distress in the first week of life who go on to develop BPD. We are also the first group to show that, in neonatal mice, IL-17A is required for chronic LPS induced hypoalveolarization and that neonatal lung γδ T cells are the major source of IL-17a after acute and chronic LPS exposure.

γδ T cells home to mucosal surfaces of the lung and other organs where they produce IL-17a and promote inflammation, while they also contribute to protective antimicrobial immunity (47, 69). IL-17-producing γδ T cells can elicit damage after infiltrating or accumulating in target tissues and promote inflammatory diseases in various organs, including contribute to hyperinflammation in chronic granulomatous disease in the lung (62, 69). The development of mouse γδ T cell IL-17-producing capacity is believed to be restricted to fetal and perinatal life, and is dependent on oxidative metabolism (46, 70). In contrast, acquisition of IL-17-producing fate by human γδ T cells requires peripheral activation under inflammatory conditions (71, 72). Cord blood naïve γδ T cells from placentas of normal, full term deliveries can differentiate into IL-17+IFN-γ- γδ T cells with a cytotoxic potential in the presence of IL-23 and TCR signaling (72). Peripheral blood γδ T cells from preterm infants show a propensity for heightened pro-inflammatory responses compared to term-born infants (24). Our findings that lung γδ T cells are the major source of IL-17a after acute and chronic LPS exposure and that TCRd-/- neonatal mice and mice treated with anti-IL-17a antibody are protected from chronic LPS-induced pulmonary inflammatory cell infiltration and hypoalveolarization are consistent with the notion that LPS induced an IL-17-driven pro-inflammatory phenotype in these cells. Further, our findings that both IL-17A and IL-23 are higher in tracheal aspirates from preterm infants who go on to develop BPD implicate this pathway in BPD pathogenesis.

NKG2D is a novel C-type lectin-like receptor expressed by γδ T cells, NK cells, NKT cells and CD8+ T cells (51). Upon binding to ligands, NKG2D activation promotes cytotoxic and proinflammatory IL-17a responses (51–53). NKG2D regulates Th1 and Th17 differentiation in mice (53). Engagement of NKG2D by its ligands, such as Rae-1, expressed on the surface of stressed cells, induces expansion of IL-17a-producing γδ T cells and genetic ablation or antibody blockade of NKG2D attenuates this effect in mice (73). A number of pro-inflammatory cytokines, including IL-7 induce NKG2D expression (74). IL-7 also promotes selective IL-17A differentiation in mouse and human γδ T cells (48), however, the requirement of NKG2D for this effect unknown. In this manuscript, we used anti-NKG2D antibody and Nkg2d-/- mice to show that NKG2D is required for chronic LPS-induced IL-17a expression, pulmonary inflammation and hypoalveolarization. However, we have not determined yet if this effect is dependent on blocking NKG2D only in γδ T cells or if other cells, such as NK cells are involved. NKG2D mediates NK cell hyperresponsiveness and development of lung emphysema in response to cigarette smoke- induced NKG2D ligand expression (75, 76). NK cells and NKG2D receptor signaling are also required for ischemia-reperfusion acute lung injury in mice (77). Our future studies will examine the contribution of NKG2D signaling in other innate lymphocytes to neonatal LPS-induced lung injury. Further, we will examine if selective TLR4 signaling in γδ T cells contributes to the neonatal LPS-induced inflammation and injury (49).

To confirm the activation of the NKG2D pathway in our model, we showed that LPS induced neonatal lung mRNA expression of NKG2D and the NKG2D ligands Rae-1 and MULT1, as well as the expression of the DNA damage factor p53. We also showed that γδ T cells upregulate NKG2D expression in response to acute and chronic LPS exposure. However, we did not investigate if other cell populations such as NK cells, also express NKG2D. We focused on γδ T cells as our data indicated that they are the main source of IL-17a in neonatal lungs following acute and chronic LPS exposure. We also did not examine the specific cell populations that upregulate expression of NKG2D ligands and cell stress factors. In a future study we plan to comprehensively identify the various cell types that upregulate expression of the NKG2D receptor and its ligands, and the induced cell stress pathways during acute and chronic LPS exposure. We also plan to investigate the interactions between specific NKG2D ligands and receptor expressing cells. Potential sources for NKG2D ligand expression are epithelial, endothelial cells, as well as myeloid cells. In a mouse model of pulmonary ischemia-reperfusion injury, Rae-1 expression is increased in epithelial and endothelial cells, and MULT1 was increased in epithelial cells (77). LPS induces NKG2D ligand expression in macrophages and dendritic cells (78).

Various mechanisms direct mouse γδ T cell activation and IL-17 differentiation. Due to their “pre-programmed” fate, mouse γδ T cells possess an “innate-like” mode that induces IL-17 differentiation upon exposure to pathogen-associated molecular patterns such as LPS driven by IL-1β and IL-23 produced by myeloid cells (34, 79). Additionally, some γδ T cell populations in mice and humans express toll-like receptor 4 (TLR4), a receptor for LPS, and signaling through TLR4 induces γδ T cell proliferation and IL-17 expression (49). An alternative mechanism of activation of NKG2D+IL-17a+ γδ T cells is by endogenous lipids presented by B-1a cells during viral infection (80).

In this study, we detected mRNA of the human NKG2D ligands MICA and MICB in in tracheal aspirates from premature infants mechanically ventilated for respiratory distress in the first week of life. While the number of samples was small and only samples from patients who went on to develop BPD were available and tested, we found a significant positive correlation between MICA mRNA levels and median FiO2, a risk factor for BPD development. One important point to consider is the capacity of cell-bound or secreted NKG2D ligands to persistently engage NK cells, cause NKG2D downregulation and suppress NK cell function, thus dampening pathology (81–83).

In this study, we used a mouse model of BPD with chronic early-life exposure to LPS. We identified IL-17a+ γδ T cells and NKG2D signaling as primary drivers of acute and chronic neonatal LPS exposure-induced lung immunopathology. Additionally, using tracheal aspirates of premature infants we showed higher levels of IL-17A and its upstream regulator IL-23 in infants who go on to develop BPD compared to infants who do not develop BPD. However, we have not determined the source of IL-17A in the human tracheal aspirates. A crucial point to consider are species-specific differences in γδ T cell phenotype and fate determination (46, 71, 72, 84). Thus, in addition to defining the mechanisms by which NKG2D+IL-17a+ γδ T cells contribute to LPS-induced BPD-like pathology in mice, a careful evaluation of these cells in human preterm infant lungs and airways should be completed, before broader implications to human disease are considered.

Exposures associated with preterm birth that contribute to BPD development are likely multifactorial. For example, cumulative oxygen exposure is a risk factor for BPD development (85). Hyperoxia also induces inflammation, cellular stress, and increased expression of ligands for activating receptors such as NKG2D (86–88). Future studies will examine the contribution of IL-17a+ γδ T cells and NKG2D signaling in hyperoxia-induced pulmonary inflammation.

In summary, we have provided a detailed *in vivo* analysis of IL-17a-driven pulmonary inflammation and impaired alveolar development after neonatal LPS exposure. Our studies demonstrate that IL-17a-producing γδ T cells and NKG2D signaling mediate neonatal LPS induced inflammatory cell infiltration and hypoalveolarization. Improved understanding of the mechanisms regulating inflammation and alveolar development could lead to new insights into the pathogenesis of BPD and lead to new therapeutic interventions.

## Data availability statement

The original contributions presented in the study are included in the article/supplementary materials. Further inquiries can be directed to the corresponding author.

## Ethics statement

The studies involving humans were approved by University of Michigan Institutional Review Board. The studies were conducted in accordance with the local legislation and institutional requirements. Written informed consent for participation in this study was provided by the participants’ legal guardians/next of kin. The animal study was approved by University of Michigan Committee on Use and Care of Animals. The study was conducted in accordance with the local legislation and institutional requirements.

## Author contributions

TC and AP conceived and designed the experiments, analyzed the data, and wrote the paper. TC, AB, Y-JZ, and CA performed the experiments, collected samples, interpreted data, edited and reviewed the manuscript. All authors contributed to the article and approved the submitted version.
